# Silent inflammation: a hidden cause of liver fibrosis

**DOI:** 10.3389/fphar.2026.1676534

**Published:** 2026-01-23

**Authors:** Hans-Theo Schon, Ralf Weiskirchen

**Affiliations:** Institute of Molecular Pathobiochemistry, Experimental Gene Therapy and Clinical Chemistry (IFMPEGKC), RWTH University Hospital Aachen, Aachen, Germany

**Keywords:** chronic low-grade inflammation, dietary modifications, liver fibrosis, MASLD, obesity

## Abstract

Systemic inflammation induced by adipose tissue is common in obese individuals and is often overlooked due to its subclinical nature. The constant secretion of proinflammatory factors shifts the balance toward inflammation, affecting the body’s homeostasis and facilitating the development of various chronic diseases. In the liver, proinflammatory markers, free fatty acids (FFAs), and the hormone leptin, all of which originate from adipose tissue, trigger an inflammatory response that favors fibrogenesis. Conversely, serum levels of proinflammatory factors can be used to assess both the risk of liver fibrosis and the effectiveness of treatment. Their application is straightforward due to their non-invasive nature, but it is important to confirm their reliability in future investigations. Moreover, dietary approaches to therapy, along with physical activity, deserve more attention as their effectiveness has frequently been demonstrated and they are recommended by official guidelines. The focus on reducing body weight through fat loss is especially crucial. To enhance the quality and value of dietary strategies in therapy, it is also necessary to refine and expand their potential.

## 1 Introduction

Obesity continues to increase globally, posing a significant threat to public health. In 2018, the obesity rate was 42.4% among the US population (Boutari and Mantzoros, 2022), and it is projected that by 2035, 24% of the global population will be obese (Yang et al., 2024). Adipose tissue, besides storing fat, acts as an endocrine organ, releasing factors such as tumor necrosis factor (TNF)-α and interleukin (IL)-6 (Katsarou et al., 2020; Lonardo and Weiskirchen, 2025). These factors contribute to chronic low-grade inflammation (CLGI), also known as silent inflammation (Zhu et al., 2022), affecting various organs in the body (Saltiel and Olefsky, 2017).

In the liver, FFAs are stored in hepatocytes as triglycerides (Katsarou et al., 2020). Most FFAs come from adipose tissue, are synthesized in the liver, or are derived from dietary sources (Carvalho-Gontijo et al., 2022). Over time, excessive amounts of these fats overwhelm hepatocytes, leading to the production of lipotoxic molecules. These toxic lipids cause oxidative stress, mitochondrial dysfunction, and endoplasmic reticulum (ER) stress (Lee and Friedman, 2022), ultimately resulting in damage to hepatocytes. In response to injury, hepatocytes release damage-associated molecular patterns (DAMPs) as danger signals into the extracellular space. These signals trigger sterile inflammation by activating immune cells such as Kupffer cells (KCs) and hepatic stellate cells (HSCs) (Ignat et al., 2020; Hammerich and Tacke, 2023). KCs, the liver’s resident macrophages, and other immune cells secrete proinflammatory cytokines like TNF-α, IL-6, and IL-1β, contributing to the inflammatory state (Vesković et al., 2024). Once activated, HSCs can transdifferentiate into myofibroblasts, leading to fibrosis (Horn and Tacke, 2024; LeFort et al., 2024; Lonardo and Weiskirchen, 2025). In fibrosis, continuous deposition of extracellular matrix (ECM) components by HSCs results in increased scarring and subsequent loss of liver function (Kershenobich Stalnikowitz and Weissbrod, 2003; Carvalho-Gontijo et al., 2022; Charan et al., 2023).

The majority of studies focus solely on the impact of inflammation within the liver, such as that induced by hepatotropic viruses or toxins like alcohol, while systemic inflammation is often overlooked as a potential contributor. Cytokines like IL-6, TNF-α, and IL-1β, which are typically elevated in CLGI, play a role in hepatocyte injury and the activation of KCs and HSCs. As a result, these elements serve as crucial connections between systemic inflammation and hepatic fibrogenesis.

Given CLGI’s potential to contribute to inflammatory and fibrogenic processes in the liver, as well as its ability to sustain chronic liver inflammation, it is crucial to recognize its significant role as a driver of fibrosis, particularly in terms of early detection and intervention. Early screening for CLGI could help prevent the progression of fibrogenesis to advanced stages, which are more challenging to treat. The objective of this review is to raise awareness of the impact of CLGI on liver fibrosis and to advocate for non-invasive testing for this systemic condition, which may require additional specific tests for liver fibrosis. However, with the increasing rates of obesity and the subtle onset of symptoms, further research on the relationship between silent inflammation and hepatic fibrosis is necessary. This review aims to present the current understanding of silent inflammation as a driver of fibrosis, highlighting potential diagnostic methods and treatment options.

## 2 Mechanisms of fibrogenesis in the liver

The cascade that ultimately leads to liver fibrosis is complex and initiated by the detection of components from within or outside the liver. These components can trigger a continuous inflammatory response, which is the main driver in fibrogenesis (Del Campo et al., 2018; Carvalho-Gontijo et al., 2022). Ongoing damage or death of hepatocytes, for example due to lipotoxicity, can result in mitochondrial dysfunction and the production of reactive oxygen species (ROS) (Bence and Birnbaum, 2021; Lonardo and Weiskirchen, 2025). Neutrophils engulf the remains of apoptotic cells and recruit more granulocyte precursors to the damaged site, worsening inflammation through the release of cytokines and chemokines (Carvalho-Gontijo et al., 2022). Released hepatocyte components act as DAMPs, which are recognized by nucleotide-binding oligomerization domain-like receptors (NLRs) found on the surface and inside of immune or parenchymal cells, mainly KCs and HSCs (Katsarou et al., 2020; Taru et al., 2024). As a result, the release of TNF-α, IL-1β, and IL-18 further contributes to the proinflammatory response (Del Campo et al., 2018; Katsarou et al., 2020; Charan et al., 2023; Taru et al., 2024).

**FIGURE 1:**
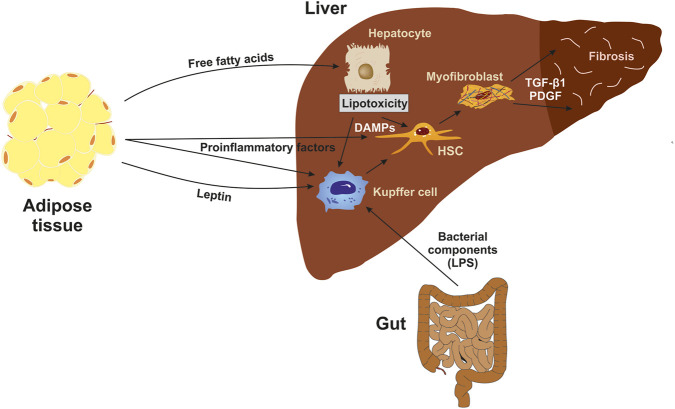
Influence of Adipose Tissue-Derived Factors on Hepatic Fibrogenesis. Free fatty acids lead to lipotoxicity in hepatocytes, which may result in apoptosis. Damage-associated molecular patterns (DAMPs) released from injured hepatocytes activate Kupffer cells (KCs) and hepatic stellate cells (HSCs). Proinflammatory factors from adipose tissue also activate KCs and HSCs. The hormone leptin and bacterial components from the gut may also activate HSCs. Once activated, HSCs transdifferentiate into myofibroblasts, which release profibrogenic cytokines such as transforming growth factor-β1 (TGF-β1) and platelet-derived growth factor (PDGF), thereby promoting fibrosis.

In summary, ROS, apoptotic bodies, profibrogenic cytokines, and proinflammatory cytokines such as TNF-α, IL-1β, IL-6, and IL-18 contribute to the activation of HSCs (Del Campo et al., 2018; Tanwar et al., 2020; Hammerich and Tacke, 2023) (Figure 1).

Upon activation, HSCs transition from a quiescent state to myofibroblasts (Hammerich and Tacke, 2023), which are crucial for producing ECM components (Del Campo et al., 2018), such as type I and type III collagens (Parola and Pinzani, 2019). They also secrete cytokines like Transforming growth factor-β1 (TGF-β1) (Ignat et al., 2020) and platelet-derived growth factor (PDGF) (Katsarou et al., 2020). TGF-β1, PDGF, and type I collagen are significant indicators of hepatic fibrosis, with increased expression observed in the liver (Vesković et al., 2024). The enhanced liver fibrosis (ELF) score includes ECM markers PRO-C3 and PRO-C6 for collagen formation, as well as C3M for type III collagen degradation (Simbrunner et al., 2022), facilitating the identification of individuals with non-alcoholic fatty liver disease (NAFLD) and advanced fibrosis (Simbrunner et al., 2022). These markers are also useful for evaluating the relationship between systemic inflammation and various stages of chronic liver disease. The following chapter will describe the characteristics of CLGI on hepatic fibrogenesis.

## 3 Principles of silent inflammation

CLGI exhibits several distinctive characteristics that set it apart from acute inflammation. Firstly, this particular type of inflammation is persistent and does not diminish over time (van de Vyver, 2023). Additionally, the absence of discernible symptoms is notable and the disease process persists in a subclinical state (van de Vyver, 2023). However, the presence of inflammatory markers can be detected at any time by measurement, even if they are only present in low concentrations. This establishes an imbalance between proinflammatory and anti-inflammatory markers (Kadatane et al., 2023) and leads to the prolonged activation of mononuclear cells, including macrophages and lymphocytes (Tanwar et al., 2020). Furthermore, CLGI has been linked to metabolic diseases, such as adiposity, metabolic syndrome, and type 2 diabetes mellitus (T2DM), as well as the aging process (Zotova et al., 2023).

In contrast, acute inflammation is a defensive reaction aimed at counteracting damage inflicted by pathogens, toxins, and burns (Maddipati, 2024). Its primary signs include erythema (rubor), warmth (calor), tumefaction (tumor), and pain (dolor) at the site of damage (Ansar and Ghosh, 2016). Damaged cells release DAMPs, which rapidly recruit neutrophils, a type of polymorphonuclear leukocyte, to the site of injury (Tanwar et al., 2020). This process typically results in a significant increase in the number of neutrophils in the blood, known as neutrophilia (Ansar and Ghosh, 2016). Treatment approaches vary. In the case of bacterial infections, the administration of antibiotics might be indicated as a treatment modality.

Given that CLGI is not confined to specific regions but involves the body as a whole (van de Vyver, 2023), it also affects the body’s homeostasis. This, in turn, facilitates the development of various chronic diseases (Tristan Asensi et al., 2023) and CLGI has been identified as a crucial factor underlying metabolic disorders, including obesity, diabetes, metabolic syndrome, and others (Margină et al., 2020). In addition, CLGI has also been linked to other pathologies, such as cardiovascular disease, cancer, and neurodegenerative disorders (Zhou et al., 2021).

Currently, there are no widely accepted biomarkers for identifying CLGI or distinguishing it from acute inflammatory responses (Minihane et al., 2015; Furman et al., 2019; Tristan Asensi et al., 2023). However, research suggests that the biomarkers commonly used, in scientific studies fall into categories such as soluble mediators, including cytokines and chemokines, and acute-phase proteins like C-reactive protein (CRP) and fibrinogen (Tristan Asensi et al., 2023). White blood cells and granulocytes are also frequently utilized as biomarkers. Another approach is the aggregated inflammation score (INFLA-score), which quantitatively assesses CLGI by incorporating CRP, white blood cell count, platelet count, and the ratio of granulocytes to lymphocytes (Cheng et al., 2024). Additionally, studies have explored the use of adhesion molecules, such as vascular cell adhesion molecule-1 (VCAM-1) and intercellular adhesion molecule-1 (ICAM-1), as well as adipokines, primarily adiponectin (Minihane et al., 2015).

**FIGURE 2:**
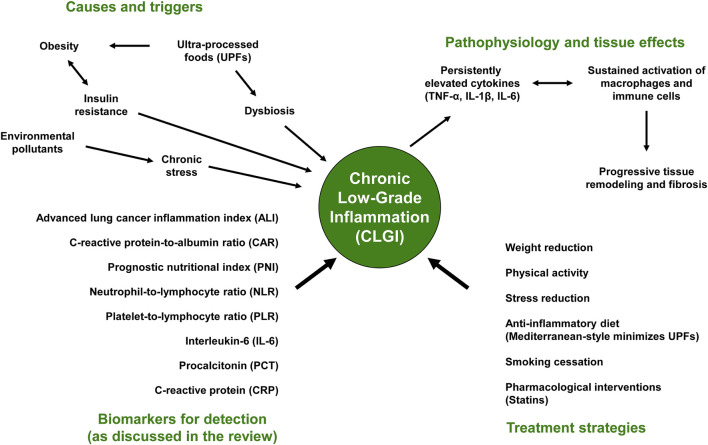
Chronic Low-Grade Inflammation (CLGI): A Multi-Systemic Perspective. The consumption of ultra-processed foods (UPFs), along with obesity, insulin resistance, environmental pollutants, and dysbiosis, has been shown to trigger CLGI. CLGI, in turn, leads to the release of proinflammatory cytokines like TNF-α, IL-1β, and IL-6, which activate macrophages and immune cells. This continuous activation of macrophages and immune cells initiates tissue remodeling and fibrosis. Biomarkers used for detection include C-reactive protein (CRP), procalcitonin (PCT), interleukin-6 (IL-6), and the neutrophil-to-lymphocyte ratio (NLR). Treatment strategies involve a comprehensive approach, including weight management, physical activity, stress reduction, an anti-inflammatory diet, smoking cessation, and pharmaceutical interventions.

The etiology of CLGI is multifactorial (Furman et al., 2019), with other diseases known to be associated with CLGI, including metabolic syndrome, T2DM, cardiovascular disease (CVD), and metabolic dysfunction-associated steatotic liver disease (MASLD) (Kadatane et al., 2023). All of these diseases share a common trait: they are perpetuated by inflammatory markers, with physical inactivity and obesity, particularly visceral adipose tissue (VAT), being the most prominent contributing factors (Burini et al., 2020). This highlights the crucial role of physical activity in preventing systemic inflammatory responses. Additional causal factors include the consumption of low-quality food, such as highly processed foods (Kadatane et al., 2023), which can negatively impact the health and diversity of gut bacteria, ultimately leading to gut dysbiosis (Margină et al., 2020). Cell senescence with aging accompanied by inflammaging (Zhou et al., 2021; Terkawi et al., 2022), environmental or industrial pollutants, and psychological stress (Furman et al., 2019) are also implicated in the development of CLGI (Figure 2).

In contrast to acute inflammation, which is mainly initiated by infections and resolves after the removal of the pathogen, CLGI is not triggered by infection. Therefore, CLGI can be defined as a sterile inflammation that does not resolve but persists (Furman et al., 2019).

The next paragraph will outline the role of CLGI in hepatic fibrogenesis and discuss several commonly used markers for detecting liver fibrosis.

## 4 How silent inflammation boosts hepatic fibrosis

Meanwhile, the correlation between metabolic dysregulation resulting from obesity and persistent hepatic inflammation has been substantiated (Katsarou et al., 2020). The presence of abundant VAT has been demonstrated to be a source of cytokines, such as TNF-α and IL-6, as well as adipokines, including leptin and adiponectin. This contributes to a proinflammatory environment, resulting in CLGI and insulin resistance in local areas and various organs (Lonardo and Weiskirchen, 2025). In addition to inducing insulin resistance in the liver, the aforementioned cytokines can also cause both fat storage and fibrosis (Katsarou et al., 2020). According to Nati and colleagues (Nati et al., 2022), in conjunction with circulating FFAs, these factors can activate KCs. The spread of inflammation from fat tissue, especially from VAT, to the liver is linked to non-alcoholic fatty liver disease (NAFLD, now known as MASLD) and has been shown to exacerbate non-alcoholic steatohepatitis (NASH, now known as MASH) in murine models (Wang et al., 2021). In addition to proinflammatory cytokines, KC activation and further macrophage recruitment are also driven by lipotoxicity and bacterial components from the gut (Nati et al., 2022).

Distinguishing between KCs and macrophages is possible through the expression of specific markers. For instance, KCs do not express chemokine (C-X_3_-C motif) receptor 1 (CX_3_CR1) while macrophages do (Koyama and Brenner, 2017). However, both cell types secrete high levels of TGF-β1, thereby contributing to hepatic fibrogenesis (Koyama and Brenner, 2017). Furthermore, they have been observed to attract a variety of immune cells to the site, including monocytes, neutrophils, and B- and T-lymphocytes (Nati et al., 2022). The recruitment of these immune cells is facilitated by chemokines, such as the C-C motif ligand 2 (CCL2), which are released by activated KCs (Nati et al., 2022). Moreover, KCs, together with hepatocytes and adipose tissue, are the predominant sources of TNF-α, which has been shown to impede the process of apoptosis in primary cultured HSCs, thereby promoting their survival and proliferation (Katsarou et al., 2020). In summary, activated KCs play a pivotal role in amplifying inflammatory and fibrogenic processes in the liver.

The hormone leptin is produced in adipose tissue and regulates appetite and the body’s energy consumption through the nervous system. In cases of obesity, there is an increase in leptin levels due to higher fat stores. However, the brain does not respond appropriately to this change, leading to a condition known as leptin resistance. High levels of leptin can stimulate KCs to release proinflammatory factors, which then affect HSCs and promote fibrosis (Lonardo and Weiskirchen, 2025). One of these factors is TGF-β1 (Katsarou et al., 2020), which is crucial in the activation of HSCs by leptin, contributing to liver fibrosis (Wang et al., 2009). Studies in rats lacking the leptin receptor have shown resistance to liver fibrosis induced by carbon tetrachloride (Koyama and Brenner, 2017).

In addition to well-established markers of inflammation, such as high-sensitivity C-reactive protein (hsCRP) (Kurt et al., 2025) and the proinflammatory cytokines IL-6 (Sharif et al., 2021; Vázquez-Lorente et al., 2025) and TNF-α (Vázquez-Lorente et al., 2025; Ramos-Nino, 2025), which are indicative of CLGI, only a limited number of studies have examined the direct relationship between systemic inflammation and MASLD or fibrosis.

One study examined the correlation between various systemic inflammation markers and MASLD (Qiu et al., 2025). While many biomarkers showed a significant association with MASLD risk, only five were found to have a positive or negative correlation: The advanced lung cancer inflammation index (ALI), the C-reactive protein-to-albumin ratio (CAR), and the prognostic nutritional index (PNI) were linked to a higher risk of MASLD, while the neutrophil-to-lymphocyte ratio (NLR) and the platelet-to-lymphocyte ratio (PLR) were negatively associated with MASLD risk (Qiu et al., 2025). Therefore, elevated levels of ALI, CAR, and PNI, along with decreased levels of NLR and PLR, may indicate an increased risk of MASLD.

In another investigation serum markers indicative of systemic inflammation (SI) were examined for their potential association with hepatic fibrogenesis and the turnover of the ECM in individuals with advanced chronic liver disease (ACLD) (Simbrunner et al., 2022). The findings of the study demonstrated that IL-6, procalcitonin (PCT), and CRP levels were independently associated with the ELF score, as well as with PRO-C3 and PRO-C6, which indicate collagen formation. Furthermore, the three SI markers were independently associated with TIMP-1. IL-6 and CRP were also linked to C3M, which, along with TIMP-1, represent matrix turnover. Additionally, a statistically significant correlation was observed between all three SI biomarkers and the proportionate area of α-SMA as determined through histological examination of hepatic tissue. In summary, the study’s findings suggest a correlation between systemic inflammation and the processes of hepatic fibrogenesis and ECM turnover in patients suffering from ACLD (Simbrunner et al., 2022) (Table 1).

**TABLE 1:** Markers indicative of systemic inflammation and their associations.

Systemic marker of inflammation	Components	Sample source	Interpretation	Association with MASLD risk	Association with hepatic fibrosis	Association with matrix formation	Association with matrix turnover	References
Advanced lung cancer inflammation index (ALI)	Body mass index (BMI), serum albumin, and NLR	Height, weight + blood serum + blood cells	Lower ALI → high inflammation; heightened ALI → low inflammation	Heightened risk	ND	ND	ND	Qiu et al. (2025)
C-reactive protein-to-albumin ratio (CAR)	C-reactive protein (CRP), albumin	Blood serum	Low CAR → low inflammation; high CAR → inflammation	Heightened risk	ND	ND	ND	Qiu et al. (2025)
Prognostic nutritional index (PNI)	Serum albumin and total lymphocyte count	Blood serum + blood cells	Low PNI → high inflammation; high PNI → low inflammation	Heightened risk	ND	ND	ND	Qiu et al. (2025)
Neutrophil-to-lymphocyte ratio (NLR)	Absolute neutrophil and absolute lymphocyte counts	Blood cells	High NLR → inflammation	Reduced risk	ND	ND	ND	Qiu et al. (2025)
Platelet-to-lymphocyte ratio (PLR)	Platelet and lymphocyte counts	Blood cells	Low PLR → no pro-inflammatory, pro-thrombotic state; high PLR → inflammation	Reduced risk	ND	ND	ND	Qiu et al. (2025)
Interleukin-6 (IL-6)	Polypeptide	Blood serum	Modest, chronic elevation → CLGI	ND	Associated with ELF score and area of α-SMA	Associated with PRO-C3 and PRO-C7	Associated with TIMP-1 and C3M	Simbrunner et al. (2022)
Procalcitonin (PCT)	Glycoprotein	Blood serum	Typically very low or undetectable	ND	Associated with ELF score and area of α-SMA	Associated with PRO-C3 and PRO-C7	Associated with TIMP-1	Simbrunner et al. (2022)
C-reactive protein (CRP)	Pentameric protein	Blood serum	<10 mg/L→ normal or slightly elevated (obesity/diabetes)	ND	Associated with ELF score and area of α-SMA	Associated with PRO-C3 and PRO-C7	Associated with TIMP-1 and C3M	Simbrunner et al., (2022)

The table presents eight markers indicative of systemic inflammation, including their composition, structure, source, interpretation, and their association with metabolic dysfunction-associated steatotic liver disease (MASLD) risk, hepatic fibrogenesis, matrix formation and matrix degradation. The last column lists the corresponding study. ND, indicates that it was not determined.

Among the markers mentioned above, which are also listed in Table 1, NLR is the optimal choice for clinical use. This biomarker reflects the relationship between the innate immune system, represented by neutrophils that cause tissue damage, and the adaptive immune system, represented by lymphocytes, whose numbers are often reduced in progressive fibrosis. As a result, patients with MASLD can have significantly elevated levels of NLR, which may indicate active fibrosis (Yang et al., 2025). Additionally, the NLR increases with the severity of hepatic fibrosis (Shavakhi et al., 2022), helping to stratify patients for further medical evaluations, such as elastography. Since NLR can be obtained from a standard complete blood count (CBC), the test is a readily available and cost-effective alternative to testing for IL-6 or PCT.

In conclusion, markers of systemic inflammation not only serve as indicators but also as potential therapeutic targets. Successfully reducing adipose tissue can decrease pressure on the liver, leading to significant improvements. The following section will address both diagnostic and therapeutic aspects.

## 5 Significance for diagnostics and therapy

The treatment of liver diseases involves a comprehensive approach that includes addressing etiological, pharmacotherapeutic, and nutritional aspects, as well as implementing lifestyle changes and prevention measures. A fundamental therapy principle is the elimination of causative agents, such as viruses or alcohol (Gan et al., 2025).

Vaccination against hepatotropic viruses is crucial for preventing infections, while abstaining from alcohol can improve prognosis, reduce hepatic injury, and slow the progression of fibrosis.

Dietary modifications and increased physical activity are essential for treating MASLD, aiming to achieve weight loss to improve insulin sensitivity and counteract metabolic syndrome and T2DM (Gan et al., 2025). The recent approval of Resmetirom by the U.S. Food and Drug Administration (FDA) for the treatment of NASH in adults with moderate or advanced hepatic fibrosis highlights the lack of other available drugs for this condition. Therefore, there is a need to focus on utilizing non-invasive diagnostic tools for early disease identification and treatment options (Gan et al., 2025). Biomarkers of SI, although based on associations rather than causal relationships, have the potential to address this need. Further research could validate their practicality and expand their application.

A focus on lifestyle factors, including diet and exercise, as the primary treatment strategy for MASLD or fibrosis (Katsarou et al., 2020; Ortiz-López et al., 2022; Guariglia et al., 2023) has been shown to be effective, when individuals adhere to the recommended guidelines. For instance, a weight reduction of 3–5 percent over a year can reverse liver steatosis, while a reduction of 7–10 percent may alleviate metabolic dysfunction-associated steatohepatitis (MASH). Moreover, a weight loss exceeding 10 percent can lead to regression of fibrosis (Gan et al., 2025). The emphasis on dietary factors is also evident in dietary recommendations, such as the European MASLD guidelines, which promote a Mediterranean-style diet, consisting mainly of unprocessed or slightly processed foods (Lonardo and Weiskirchen, 2025).

Two cases illustrate the effectiveness of a ketogenic diet. In both studies daily carbohydrate intake was restricted to induce ketosis. In one study, participants consumed less than 30 g of carbohydrates per day for 90 days. The main outcomes of this study showed a reversal of diabetes, as indicated by a reduction in hemoglobin A1C (HbA1_C_) and weight loss, among other benefits (Walton et al., 2019). The second study utilized a very low-calorie ketogenic diet (VLCKD), with carbohydrate intake ranging from 20 to 50 g per day. The aim was to evaluate the impact of VLCKD on inflammatory markers (white blood cell count, platelet count, and hsCRP serum levels) associated with low-grade inflammation in obese individuals. The results revealed a decrease in low-grade inflammation, as well as an improvement in hepatic steatosis and fibrosis (De Nucci et al., 2023).

A meta-analysis of 37 randomized controlled trials (RCTs) in participants suffering from MASLD or MASH has shown that the Mediterranean diet reduced weight, body mass index (BMI), waist circumference, and alanine aminotransferase (ALT) levels, compared to the control group (Arita et al., 2025). The authors concluded that the Mediterranean diet, when combined with aerobic exercise and potentially supplemented with resistance training, is linked to weight loss and improved liver health. They also emphasized the importance of promoting lifestyle interventions in managing MASLD and MASH.

In an RCT involving 294 individuals with dyslipidemia and abdominal obesity, the effects of following a Mediterranean diet along with three to four cups of green tea, a Mankai shake daily, physical activity, and reduced processed meat intake were examined (Yaskolka Meir et al., 2021). The results revealed that the intervention led to a 50% reduction in intrahepatic fat levels in participants after an 18-month period, nearly double the reduction compared to following a Mediterranean diet alone or a healthy dietary approach alone.

Additionally, several naturally occurring compounds were examined for their potential benefits on fibrosis, with a particular focus on the polyphenols resveratrol, silymarin, and curcumin (Roehlen et al., 2020). Polyphenols are well-known for their antioxidant qualities and have been demonstrated to impede oxidative stress, trigger the oxidation of FFAs, and ameliorate insulin resistance (Guariglia et al., 2023). Moreover, curcumin can reduce inflammation, exhibits anti-fibrotic activity, and reestablishes the balance in the microbiota. The examination of these effects of curcumin was already part of clinical trials conducted on individuals suffering from MASLD in order to evaluate liver condition (Guariglia et al., 2023).

Furthermore, probiotics (microorganisms), prebiotics (fiber, their food), and biogenics (prebiotics, probiotics and their products) have also gained attention, due to their influence on the intestinal microbiota, immunity, and the gut-liver axis (Nagashimada and Honda, 2021; Guariglia et al., 2023).

Interestingly, ghrelin, a gastric peptide hormone, has been shown to possess a dual function. On the one hand, the peptide can be used as a marker for NASH, while also being a possible target in NASH therapy. During low-grade inflammation, ghrelin impedes the polarization of KCs or macrophages into the M1-phenotype, thereby exerting an anti-inflammatory effect and inhibiting NASH (Yin et al., 2021). In addition to the nutritional strategies, the application of naturally occurring compounds has also been shown to have significant effects on liver health and overall wellbeing. Therefore, further investigation is warranted.

## 6 Concluding remarks

Apart from Resmetirom, which has been approved for use in the U.S., there are currently no other pharmaceutical agents available for the treatment of liver fibrosis. Therefore, it is important to consider the contribution of CLGI to liver fibrosis. This systemic inflammation can be managed quickly and successfully. Multiple biomarkers have been identified that are suitable for detecting CLGI. Furthermore, therapeutic interventions that result in weight loss can have promising effects on overall health, particularly on liver health. Consequently, it is appropriate to prioritize diagnostic tests that indicate systemic inflammation in the risk assessment of fibrosis. However, it is also important to intensify research on biomarkers of systemic inflammation and further solidify our current knowledge of their role in hepatic fibrosis.
